# Eating Dirt

**DOI:** 10.3201/eid0908.030033

**Published:** 2003-08

**Authors:** Gerald N. Callahan

## Abstract

Please This earth is blessed Do not play in it Sign on the wall of El Santuario de Chimayo, New Mexico

This place feels old beyond human recollection. The carvings and paintings were surely done by human hands, but no one remembers whose hands those were. The work is striking, especially in the apse behind the altar. There, the colors of surrounding hills have been transferred onto nearly luminous wooden reredos full of Catholic symbolism. Above the altar hangs a most intricate ancient Christ crucified on a green cross. Overhead, the roof is held in place by massive carved wooden beams, big around as human bodies and blackened by nearly two centuries of incense and candle smoke. The air is rich with the memory of thousands of benedictions and baptisms. Threadbare trousers have polished the pews to a high varnish that this afternoon ripples with a low orange glow from dozens of votive candles burning purposefully in back of the church.

This is El Santuario de Chimayo, an old adobe-brick and stucco structure in the hills of northern New Mexico. This chapel was built in 1816, but a sanctuary has been at this site for much longer. The locals offer many legends about its origins, fanciful tales of miraculous crucifixes and Santo Niños. But the truth is buried beneath the murk of time. One thing is clear though, as beautiful as the sanctuary is and as striking as the crucifix (El Sefior de Esquipalas) above the altar is, nearly none of those in the pews today have come to see the sanctuary or the crucifix. Instead, they have come from all over the world to this place in New Mexico to eat the dirt that lies beneath the adobe floor.

According to legend, that dirt is sacred, consecrated by Christ himself. Crutches cast off by the newly healed fill the anteroom, and on some days, the line of pilgrims stretches for blocks. Some call this place the Lourdes of America, but in Chimayo the miracle can be seen each day by anyone who peers into a low-ceilinged room off the main entrance. There, a hole (the *posito*), half a meter across, pierces the floor. Beside it, someone has left a plastic spoon to aid the faithful. Beyond the spoon, beneath the opening, lies only dirt, only the deep-red dirt of Chimayo.

Most of the faithful here today have come to eat that dirt. This religious tradition is practiced, as far as I know, only at one other place—a Catholic shrine in Esquipalas, Guatemala. But pilgrims to these shrines are not the only humans who eat dirt. Nor are religious reasons the only reasons to imagine that dirt may have special powers.

## Geophagy (Eating Dirt) and Its Reasons

Other than water, what little stuff we humans have inside us is largely dirt. Admittedly, this dirt is sometimes highly processed before we receive it, but most solids that make up humans and other creatures either are now or recently were dirt (the simple stuff that stripes the outer surface of our world, the thin paste that raises us above rocks) transformed by sunlight into plants or animals. Most of us prefer the dirt we eat in the form of cows and sheep and carrots and squash and bison and sorghum. Other dirt we’d just as soon scrape from our feet and leave at the door.

But not everyone wishes to be so far removed from the stuff of mud pies and mucilage. On every continent (except, possibly, Antarctica), some of us intentionally eat dirt, and we are joined in this practice by a myriad of rats, mice, mule deer, birds, elephants, African buffalo, cattle, tapirs, pacas, and several species of primates (*1*). Most scientists consider animal geophagy “normal,” probably because most soil consumption by animals has no obvious adverse effects and is sometimes beneficial (*2*); however, some of these same scientists consider most (or all) human geophagy “abnormal.”

## Abnormal Behavior

In the United States, many of us believe that humans should only eat food. We consider the consumption of nonfood items pathological, even though we know that what people define as “food” varies dramatically by region and ethnicity. We call the pathological act of eating nonfood items pica. Pica is a disease, but a disease different from polio or smallpox. No infectious agent is obviously associated with pica. Pica is a disease only because we believe normal “undiseased” persons would not eat anything but traditional human foods; some of those who do, some of the time, are at considerable risk because of their unusual appetites.

Pathological consumption of soil, “soil pica,” is associated with several psychological abnormalities. But all ingestion of soil is not soil pica. How much soil a person has to eat to be considered ill is not known. One report described soil pica in a developmentally disabled person who regularly consumed more than 50 g of soil per day (*3*). Most of us would consider that level of geophagy at least potentially pathological, although I am not sure why.

In June 2000, the U.S. Agency for Toxic Substances and Disease Registry appointed a committee to review soil pica. The committee settled on pathological levels as consumption of more than 500 mg of soil per day but conceded that the amount selected was arbitrary (*3*). Soil consumption is defined as pathological according to the amount eaten (no normal person could possibly eat that much dirt) and the severity of health consequences (lead poisoning, parasites). Because underlying psychological or biologic abnormalities are not easy to establish, I explore only what appears to be nonpathological dirt eating in pregnant women (especially in sub-Saharan Africa), migrants from sub-Saharan cultures to other parts of the world (notably the United States), and children worldwide.

## Inadvertent Exposure

Why is it, that in spite of all the times we’ve been told not to, we still eat dirt? This is a very complex question with many possible answers. And while each proposed answer has its advocates, no single answer seems satisfactory to all—except one. Almost everyone agrees on one cause of geophagy, inadvertent consumption of air-, water-, and foodborne dirt. Contaminated food, soiled hands, and inhaled dust add soil to our diets. Children ingest considerable amounts of soil in these ways. My children did. Of course, my children also ate dirt on purpose. But child or adult, each of us inadvertently eats a little dirt every day. This dirt can pose a health threat, especially near sites of industrial contamination, but dirt we eat intentionally poses a greater challenge. Intention may indicate something biologic that drives some of us (sometimes regularly, sometimes religiously, sometimes ritually) to eat dirt.

## Tradition and Culture

For centuries, indigenous peoples have routinely used clays (decomposed rock, silica and aluminum or magnesium salts, absorbed organic materials) in food preparation. The clays were used to remove toxins (e.g., in aboriginal acorn breads); as condiments or spices (in the Philippines, New Guinea, Costa Rica, Guatemala, the Amazon and Orinoco basins of South America); and as food during famine (*4*). Clays were also often used in medications (e.g., kaolin clay in Kaopectate). But the most common occasion for eating dirt in many societies (the only occasion in some societies) is pregnancy. When sperm and egg collide, the world changes. That is obvious. But why pregnant women eat dirt is not.

Wiley and Katz (*5*) have proposed that eating clay serves different purposes during different periods of pregnancy, soothing stomach upset during morning sickness in the first trimester and supplementing nutrients (especially calcium) during the second and third trimesters, when the fetal skeleton is forming. This type of geophagy occurs most commonly in cultures of sub-Saharan Africa and their descendants (*5*). The timing of dirt ingestion and amounts consumed vary with tribes and individual persons, but soil comes consistently from certain sites. In some cultures, well-established trade routes and clay traders make rural clays available for geophagy even in urban settings. Clays from termite mounds are especially popular among traded clays, perhaps because they are rich in calcium (*5*). Whatever the underlying reason, geophagy in Africa does not appear to be a recent cultural development; it may predate *Homo sapiens.*

Women eat dirt during the first, second, or third trimester or throughout pregnancy (*5*), often throughout the day, as a supplement rather than a meal. Most commonly consumed are subsurface clays, especially kaolin and montmorillonite (*5*), 30 g to 50 g a day (sometimes much more) (*3*). However, eating dirt is not always confined to pregnant women, even among the cultures of sub-Saharan Africa (*4*), nor is it limited to tribes with little or no access to dairy-derived calcium (*5*), so these hypotheses do not adequately explain local tastes for dirt.

Soil, including kaolinitic and montmorillonitic clays, contains considerable amounts of organic material, including many live microorganisms. The human gut is the largest area of direct contact between a person and the world. Gut-associated lymphoid tissue (GALT) is a major site of T-cell differentiation and selection in adults and of intense immunologic activity (including T lymphopoiesis) in children and adults (*6*–*9*). And while it is not entirely clear why some gut-introduced antigens promote tolerance of microorganisms and others immunize against them (*10*), it is clear that immunization via the gut is a major source of immunoglobulin (Ig) A, both locally and systemically (*6*–*10*).

Regular consumption of soil might boost the mother’s secretory immune system. Monkeys that regularly eat dirt have lower parasite loads (*1*). In some cultures, clays are baked before they are eaten, which could boost immunity from previous exposures. For decades we have used aluminum salts, like those found in clays, as adjuvants in human and animal vaccines. Adjuvants are compounds that nonspecifically amplify immune response, probably because of their effects on innate defenses such as macrophages, dendritic cells, and the inflammatory response. Aluminum compounds make effective adjuvants because they are relatively nontoxic, the charged surfaces of aluminum salts absorb large numbers of organic molecules, and macrophages and dendritic cells readily phagocytose the particulates produced by the combination of the adjuvants and the organic compounds (*11*). The clays that pregnant women and others consume, which are rich in aluminum compounds, likely make at least passable immunologic adjuvants. For all these reasons, clays might act as vaccines. And the IgA antibodies produced against the associated organic antigens may appear in breast milk and have a major role in mucosal protection of newborns.

In pregnant women, this type of gut immunization might produce high levels of IgA against endemic pathogens and other antigens. All this IgA would appear shortly before birth in the breast milk and would provide protection for infants against precisely the pathogens encountered immediately after birth. Furthermore, IgA antibodies prevent attachment of bacteria and some viruses at mucosal surfaces (*12*), the major contact between the infant and the infectious world. In humans, mucosal surfaces offer the only routes of natural immunization short of wounding, and dirt would seem to offer a potent vaccine containing many endemic pathogens—no needles, no sugar-cube, no gene gun.

Eating dirt, then, rather than being abnormal, may be an evolutionary adaptation acquired over millennia of productive and not-so-productive interactions with bacteria—an adaptation that enhances fetal immunity and increases calcium, eliminates gastric upset, detoxifies some plant and animal toxins, and perhaps boosts mothers’ immunity at times when the hormones of pregnancy (*13*), factors produced by the fetus (*14*), changes in the complement system, replacement of MHC class I antigens in the trophoblast (*15*), and who knows what else suppress the mother’s natural immunologic desire to destroy her fetus—a miracle, nearly.

## Innate Tendency

My children ate dirt with surprising gusto, garden soil, road soil, leaf-mush soil, sod soil, bug-body soil—even gutter soil. As usual with my children, before I could talk them out of this behavior, they gave it up on their own—their behavior depending more on personal likes and dislikes than on my paternal concerns. I was pleased when they quit. Later I was reassured to discover from other parents that their children were just as taken with dirt as mine, some even more so. I felt less like the parent of a couple of dirt-eating, psychosis-ridden, nutritionally deprived children, even if my children were never quite “normal.”

Eating dirt appears nearly universal among children under 2 years of age. When I asked my 2-year-old daughter why she ate dirt, she just stared at me, her eyes wide open, a thick moustache of loam limning her lips. She must have decided that either what I had asked was unfathomably abstract or her answer would be far beyond my comprehension.

Soil pica has been defined as eating 500 mg to >50 g of soil per day (*3*). But the general applicability of these numbers is widely disputed (pregnant women in Africa eat far more soil than this). By inference, however, normal soil consumption must fall into the range of 0 mg to 500 mg per day per small mouth. Soils consumed by children may differ from those consumed by adults. Generally, children consume topsoils and not the deep (60 cm- to 90-cm deep) clays adults regularly consume (*5*). And children are considerably less selective in the sites they choose for dirt to eat. But why children eat dirt remains largely obscure to all but children.

Children may eat soil for the same reasons pregnant women and some animals do (*2*,*4*,*16*–*18*). Because of their rapid growth, they have special nutritional needs and surface soils may serve as supplemental nutrients; detoxification of plant or animal toxins might be accelerated by geophagy— particularly in some parts of the world; or soil components, especially clays, may relieve gastric distress. But topsoils are probably not as effective as deep clays at gastric soothing.

Among children, too, it seems eating dirt might have immunologic consequences. Maternal immunoglobulins are secreted in breast milk shortly before birth and for 1 year or more afterwards. Children often begin eating dirt a year or two after birth. As maternal immunity wanes, eating dirt might “vaccinate” children who are losing their maternal IgA, which could stimulate production of nascent immunoglobulins, especially IgA. Eating dirt might also help populate intestinal flora.

But all of this remains speculative. No clear evidence supports a biologic benefit to geophagy among children. Its frequency and distribution, though, suggest a greater biologic involvement than the simple oral obsessions of children.

## Risks of Eating Dirt

How dangerous is eating dirt? My mother was pretty certain about this—damn dangerous. Soils contaminated by industrial or human pollutants pose considerable threat to anyone who eats them. Reports abound of lead poisoning and other toxicities in children eating contaminated soils. Similarly, we do not have to look farther than the last refugee camp or the slums of Calcutta or Tijuana or Basra to find the dangers of soils contaminated with untreated human waste. But the inherent biologic danger of soil is difficult to assess. Soil unaffected by the pressures of overpopulation, industry, and agriculture may be vastly different from the soil most of us encounter routinely.

Using DNA-hybridization analyses, Torsvik et al. (*19*,*20*) found an estimated 4,600 species of prokaryotic microorganisms per gram of natural soil. Subsequent investigations, using more sophisticated techniques, found even more species (*20*), 700–7,000 g of biomass per cubic meter of soil. Soil is a considerable biologic sink, and certainly some organisms found in it are pathogenic in humans. Yet evidence of soil as a major cause of disease in humans and other animals is limited. And many reported diseases are the result of an abnormal situation, e.g., industrial pollution or untreated sewage.

Most infectious diseases acquired through eating dirt are associated with childhood geophagy, which routinely involves topsoils rather than deep clays. One recent report describes infection of two children at separate sites with raccoon roundworm (*Baylisascaris procyonis* ) (*21*). The infection resulted in severe neurologic damage to both children, and one died. The roundworm was ingested along with soil in both cases. Eating dirt can have dire consequences.

In the United States, the most common parasitic infection associated with geophagy is toxocariasis, most often caused by the worm *Toxocara canis.* Seroprevalence is 4% to 8% depending on the region, but incidence of antibodies to *T. canis* is as high as 16%–30% among blacks and Hispanics. The most common route of infection is ingestion of soil contaminated with dog or cat feces (*22*). Even though, humans are only paratenic hosts of *T. canis*, under some circumstances (though severe cases are rare), the worm can cause considerable damage (visceral larva migrans, ocular larva migrans, urticaria, pulmonary nodules, hepatic and lymphatic visceral larva migrans, arthralgias) (*22*–*24*). Toxocara eggs persist in soil for years. As with soils contaminated by human wastes, soil consumption itself does not cause toxocariasis. And studies of seroprevalence do not distinguish between infection and immunization.

Among children in Nigeria, the most common parasitic infection associated with eating dirt is ascariasis (*25*). Ascarid worms infect as many as 25% of the world’s population (more than 1.25 billion). *Ascaris lumbricoides* is the most common worm. Asymptomatic in many adults, infection is much more serious in children; intestinal obstruction is the most common symptom. Because the worms do not replicate in humans, reexposure is required to maintain infection beyond 2 years. The correlation between geophagy and helminth infection varies with different helminthes. Geissler et al. reported correlation between geophagy and ascariasis (especially caused by *A. lumbricoides*) and possibly trichuriasis but none between geophagy and reinfection with *Schistosoma mansoni, Trichuris trichiura,* or hookworm (*26*). All parasites that infest soil do not uniformly infect people who consume dirt. Nor do all who eat dirt routinely contract disease.

## Immunologic Development and Infectious Disease

Many nonhuman animals regularly eat dirt, generally without ill effects and in many cases with some benefits. Even in humans, there are few reports of infections routinely associated with geophagy by pregnant women in sub-Saharan Africa, probably because women take clays from 60 cm to 90 cm below the soil surface and, at least some of the time, they bake the clays. But these factors seem inadequate to fully account for the frequent absence of overt ill effects.

Helminth infections associated with geophagy appear to affect the frequency of inflammatory bowel diseases, which occur most often in industrialized nations. The underlying cause of these diseases may be abnormal immune response to the contents of the gut or perhaps to the gut itself (*27*). Inflammatory bowel diseases occur at much lower rates in regions where helminth infections are common. Development of normal gut-associated immune response may be aided by the presence of worms.

In studies of healthy mice, *Trichinella spiralis* prevented colitis induced with tri-nitrobenzene sulfonic acid by redirecting a primarily Th-1 response to a Th-2 response (*28*). Preliminary studies indicate that helminth infection may also alter the course of inflammatory bowel disease in humans (*29*). Soil is a rich source of parasitic worms. Studies using a number of other animals have also, at least indirectly, associated dirt and microorganisms with normal immunity.

The Environmental Protection Agency estimates that children in the United States consume, on average, 200–800 mg of dirt per day. Some children regularly consume more than their allotment. Still, that doesn’t seem like a lot of dirt. We parents have tried for years to put a stop to it. I don’t know of an instance in which anybody has succeeded in keeping children away from dirt. But animals have been successfully raised in absolutely sterile environments. Rabbits, mice, guinea pigs, and rats have been raised under such conditions (*30*,*31*). In each case, the immune system failed to develop normally. Lymph nodes and GALT did not achieve the right shape or composition and could not initiate normal immune response. Reexposure to infection later in life does not work, at least not fully. There is a window when infection drives the immune system toward its proper end. After that, mice, rats, rabbits, and guinea pigs are at the mercy of the microbial world.

Evidence suggests that the results would be the same in children. In large families, children with many older brothers and sisters are less likely to have asthma, hay fever, or eczema. West African children who have had measles are half as likely to have allergies as children who never had measles. Italian students who recovered from infection with hepatitis A had fewer and less severe allergies than fellow students who were never infected. Children with Type I diabetes (an autoimmune disease) are less likely to have had infections before their fifth birthdays than healthy children of the same age. Children raised in rural areas, especially on farms, have fewer allergies and autoimmune diseases than children raised in cities. All of these notions have been referred to as the “hygiene hypothesis” (*32*).

Children exposed a little more to the infectious face of this world seem to fare better as adults. I do not mean to say that vaccination is inappropriate. Vaccination *is,* most often, infection, and vaccinations have done more to improve childhood survival rates that any other single bit of modern medicine. Nor are water purification and sewage treatment inappropriate. Water and sewage treatment have done even more than vaccination to eliminate disease in areas where human populations have exceeded the ability of the local environment to deal with human waste and the pathogens associated with it. But, evidence indicates that infection early in life is critical for the development of normal immune systems.

Exposure-dependent development is not limited to the immune system. Animals, humans included, must be exposed to the sights, sounds, feels tastes, and smells of this septic world. When we are not, our nervous systems do not develop normally, do not rewire, expand, and contract as they must to survive (*33*). For humans, as for rabbits, there is a window in childhood when our experiences, our infections, change everything, once and for all. Inside that window, infection causes lymph nodes and GALT to enlarge and reorganize, to separate into cortices and medullae, into primary lymphoid follicles, and develop T- and B-lymphocyte–rich regions of immune competence destined to someday be germinal centers, where our defenses will muster and the real battle will be fought. This window is a defining moment, when the simplest and lowest forms of life—the dirty, the infectious, the parasitic, and the septic—alter who we are.

We do not know which childhood infections are most important, but several studies implicate mycobacterial infections. A large group of bacteria most of which cause no apparent disease, the mycobacteria, have strains that cause serious diseases (e.g., tuberculosis, leprosy). Mice injected with ovalbumin (the major protein in egg white) become allergic to ovalbumin. But mice first infected with mycobacteria and then injected with ovalbumin do not become allergic (*34*).

Early infection of children with some mycobacteria may promote strong immune systems, a normal sense of self, and a normal defense of that self. Mycobacteria are found in large numbers in dirt. And animals (probably including humans) kept from this dirt may lose the ability to recognize certain dangerous organisms as a threat, lose the ability to discriminate between self and not self, and lose the ability to distinguish the fatal from the innocuous.

## The “Age of Bacteria”

For more than 3 billion years, microorganisms, especially bacteria, have ruled earth. As Stephen Jay Gould said, “We live now in the ‘Age of Bacteria.’ Our planet has always been in the ‘Age of Bacteria’ ever since the first fossils, bacteria of course, were entombed in rocks more than three and a half billion years ago” (*35*). And bacteria have done more than any other living group to alter the character of this earth (*36*). It has been estimated that more than 10^29^ bacteria live on this planet and as many as 10^14^ live on each one of us. Through all of history, we humans have waltzed with bacteria and the rest of the microscopic world. We had no choice. Bacteria outnumber, outweigh, out-travel, and outevolve us.

That bacteria cause so many human diseases is not astounding. It is astounding that so few bacteria cause human disease. Pathogenic bacteria are merely the microscopic tip of the largest of all biologic icebergs. How fortunate, we imagine. But fortune may have little or nothing to do with our survival. Billions of years of confrontation rather than luck were likely our benefactor. Through those confrontations and those eons, nearly all of us learned to coexist peacefully. Neither humans nor microorganisms benefit from fully destroying the other. Fatal infections seem, biologically at least, shortsighted. And even a brief course of antibiotics is enough to remind us that a world without bacteria would be a poorer world. This is not a war, as it has often been described, even though we have mustered an impressive array of weapons—bactericidal cribs and mattresses, toilet cleaners and counter tops, blankets, deodorants, shampoos, hand soaps, mouthwashes, toothpastes. This is not a war at all. If it were, we would have lost long ago, overpowered by sheer numbers and evolutionary speed. This is something else, something like a lichen, something like a waltz. This waltz will last for all of human history. We must hold our partners carefully and dance well.

## Chimayo

Here beneath the old wood crucifix, as I watch the faithful leave the little chapel in Chimayo, I marvel with them at the miracle beneath this adobe floor, the same miracle buried beneath most every place human feet have trod.

## References

[1] Krishnamani R, Mahaney WC. Geophagy among primates: adaptive significance and ecological consequences. Anim Behav. 2002;59:899–915. 10.1006/anbe.1999.1376 10860518

[2] Diamond J. Dirty eating for healthy living. Nature. 1999;400:120–1. 10.1038/22014 10408435

[3] Summary report for the ATSDR Soil-Pica Workshop, Atlanta, Georgia, 2000. Available from: URL: http://www.atsdr.cdc.gov/NEWS/soilpica.html

[4] Johns T. With bitter herbs they shall eat it: chemical ecology and the origins of human diet and medicine. Tucson (AZ): University of Arizona Press; 1990.

[5] Wiley AS, Solomon HK. Geophagy in pregnancy: a test of a hypothesis. Curr Anthropol. 1998;39:532–45. 10.1086/204769

[6] Guy-Grand D, Azogui O, Celli S, Darche S, Nussenzweig M, Kourilsky P, Extrathymic T cell lymphopoiesis. J Exp Med. 2003;197:333–41. 10.1084/jem.20021639 12566417 PMC2193840

[7] Heuy Ching W, Zhou Q, Dragoo J, Klein JR. Most murine CD8^+^ intestinal intraepithelial lymphocytes are partially but not fully activated. J Immunol. 2002;169:4717–25. 12391179 10.4049/jimmunol.169.9.4717

[8] Lambolez F, Azogui O, Joret A, Garcia C, von Boehmer H, Di Santo J, Characterization of T cell differentiation in the murine gut. J Exp Med. 2002;195:437–49. 10.1084/jem.20010798 11854357 PMC2193617

[9] Poussier P, Julius M. Thymus independent T cell development and selection in the intestinal epithelium. Annu Rev Immunol. 1994;12:521–53. 10.1146/annurev.iy.12.040194.002513 8011290

[10] Mowat AM. Anatomical basis of tolerance and immunity to intestinal antigens. Nat Rev Immunol. 2003;3:331–41. 10.1038/nri1057 12669023

[11] Gupta RK. Aluminum compounds as vaccine adjuvants. Adv Drug Deliv Rev. 1998;32:155–72. 10.1016/S0169-409X(98)00008-8 10837642

[12] Noguera-Obenza M, Ochoa TJ, Gomez HF, Guerrero ML, Herrera-Insua I, Morrow AL, Human milk secretory antibodies against attaching and effacing *Eschericia coli* antigens. Emerg Infect Dis. 2002;9:545–55. 10.3201/eid0905.020441 PMC2972756 12737737

[13] Smith JL. Foodborne infections during pregnancy. J Food Prot. 1999;62:818–29. 10419281 10.4315/0362-028x-62.7.818

[14] Munn DH, Shou M, Attwood JT, Bondarev I, Conway SJ, Marshall B, Prevention of allogeneic fetal rejection by tryptophan catabolism. Science. 1998;281:1191–3. 10.1126/science.281.5380.1191 9712583

[15] Moffet-King A. Natural killer cells and pregnancy. Nat Rev Immunol. 2002;2:656–61. 10.1038/nri886 12209134

[16] Abrahams PW. The chemistry and mineralogy of three Savanna lick soils. J Chem Ecol. 1999;25:2215–28. 10.1023/A:1020861505138

[17] Gilardi JD, Duffey SS, Munn CA, Tell L. Biochemical functions of geophagy in parrots: detoxification of dietary toxins and cytoprotective effects. J Chem Ecol. 1999;25:897–922. 10.1023/A:1020857120217

[18] Johns T, Duquette M. Detoxification and mineral supplementation as functions of geophagy. Am J Clin Nutr. 1991;53:448–56. 1989412 10.1093/ajcn/53.2.448

[19] Torsvik V, Salte K, Sorheim R, Goksoyr J. Comparison of phenotypic diversity and DNA heterogeneity in population of soil bacteria. Appl Environ Microbiol. 1990;56:776–81. 2180371 10.1128/aem.56.3.776-781.1990 PMC183420

[20] Kent A, Triplett EW. Microbial communites and their interactions in soil and rhizosphere ecosystems. Annu Rev Microbiol. 2002;56:211–36. 10.1146/annurev.micro.56.012302.161120 12142496

[21] Centers for Disease Control and Prevention. Racoon roundworm encephalitis—Chicago, Illinois, and Los Angeles, California, 2000. MMWR Morb Mortal Wkly Rep. 2002;50:1153–5. 11838448

[22] Laufer M. Toxocariasis. Available from: URL: http://www.emedicine.com/ped/topic2270.htm, 2002

[23] Glickman LT, Schantz PM. Epidemiology and pathogenesis of zoonotic toxocariasis. Epidemiol Rev. 1981;3:230–50. 7030762 10.1093/oxfordjournals.epirev.a036235

[24] Kazacos KR. Visceral and ocular larva migrans. Semin Vet Med Surg (Small Anim). 1991;6:227–35. 1962007

[25] Ozumba UC, Ozumba N. Patterns of helminth infection in the human gut at the University of Nigeria Teaching Hospital, Enugu, Nigeria. J Health Sci. 2002;48:263–8. 10.1248/jhs.48.263

[26] Geissler PW, Mwaniki D, Thiong F, Friis H. Geophagy as a risk factor for geohelminth infections: a longitudinal study of Kenyan primary schoolchildren. Trans R Soc Trop Med Hyg. 1998;92:7–11. 10.1016/S0035-9203(98)90934-8 9692137

[27] Elliott DE, Li V, Blum A, Metawali A, Urban JF, Weinstock JV. Exposure to schistosoma eggs protects mice from TNBS-induced colitis. Gastrointestinal and Liver Physiology. 2003;284:385–91. 10.1152/ajpgi.00049.2002 12431903

[28] Elliott DE, Urban JF, Curtis AK, Weinstock JV. Does the failure to acquire helminthic parasites predispose to Crohn's disease. FASEB J. 2000;14:1848–55. 10.1096/fj.99-0885hyp 10973934

[29] Khan WI, Blennerhasset PA, Varghese AK, Chowdhury SK, Omsted P, Deng Y, Intestinal nematode infection ameliorates experimental colitis in mice. Infect Immun. 2002;70:5931–7. 10.1128/IAI.70.11.5931-5937.2002 12379667 PMC130294

[30] Lanning D, Sethupathi P, Rhee KJ, Zhai SK, Knight KL. Intestinal microflora and diversification of the rabbit antibody repertoire. J Immunol. 2000;165:2012. 10925284 10.4049/jimmunol.165.4.2012

[31] Paul W, ed. Fundamental immunology. New York: Lippincott Raven; 1999.

[32] Weiss ST. Eat dirt – the hygiene hypothesis and allergic disease. N Engl J Med. 2002;347:930–1. 10.1056/NEJMe020092 12239263

[33] Callahan GN. Faith, madness, and spontaneous human combustion: what immunology can teach us about self-perception. New York: St. Martins Press;2002.

[34] Zuany-Amorim C, Elzbieta S, Manilu C, Le Moine A, Brunet LR, Kemeny DM, Suppression of airway eosinophilia by killed *Mycobacterium vaccae*-induced allergen-specific regulatory T-cells. Nat Med. 2002;8:625–9. 10.1038/nm0602-625 12042815

[35] Gould SJ. Full house. New York: Harmony Books; 1996.

[36] Margulis L, Sagan D, Thomas L. Microcosmos: four billion years of evolution from our microbial ancestors. Berkeley (CA): University of California Press; 1997.

